# The community impact of the 2009 influenza pandemic in the WHO European Region: a comparison with historical seasonal data from 28 countries

**DOI:** 10.1186/1471-2334-12-36

**Published:** 2012-02-10

**Authors:** Liana Martirosyan, W John Paget, Pernille Jorgensen, Caroline S Brown, Tamara J Meerhoff, Dmitriy Pereyaslov, Joshua A Mott

**Affiliations:** 1 Netherlands Institute for Health Services Research (NIVEL), Otterstraat 118-124, P.O. Box 1568, 3500 BN Utrecht, the Netherlands; 2 Influenza & other Respiratory Pathogens, Division of Health Security, Infectious Diseases and the Environment, WHO Regional Office for Europe, Scherfigsvej 8, 2100, Copenhagen, Denmark; 3 Department of primary and community care, the Netherlands Radboud University Medical Center Nijmegen, Geert Grooteplein-Zuid 10 6525 GA, Nijmegen, the Netherlands

**Keywords:** Influenza, Human, Pandemics, Epidemiology, Community health services, Ambulatory care, Sentinel surveillance

## Abstract

**Background:**

The world has recently experienced the first influenza pandemic of the 21st century that lasted 14 months from June 2009 to August 2010. This study aimed to compare the timing, geographic spread and community impact during the winter wave of influenza pandemic A (H1N1) 2009 to historical influenza seasons in countries of the WHO European region.

**Methods:**

We assessed the timing of pandemic by comparing the median peak of influenza activity in countries of the region during the last seven influenza seasons. The peaks of influenza activity were selected by two independent researchers using predefined rules. The geographic spread was assessed by correlating the peak week of influenza activity in included countries against the longitude and latitude of the central point in each country. To assess the community impact of pandemic influenza, we constructed linear regression models to compare the total and age-specific influenza-like-illness (ILI) or acute respiratory infection (ARI) rates reported by the countries in the pandemic season to those observed in the previous six influenza seasons.

**Results:**

We found that the influenza activity reached its peak during the pandemic, on average, 10.5 weeks (95% CI 6.4-14.2) earlier than during the previous 6 seasons in the Region, and there was a west to east spread of pandemic A(H1N1) influenza virus in the western part of the Region. A regression analysis showed that the total ILI or ARI rates were not higher than historical rates in 19 of the 28 countries. However, in countries with age-specific data, there were significantly higher consultation rates in the 0-4 and/or 5-14 age groups in 11 of the 20 countries.

**Conclusions:**

Using routine influenza surveillance data, we found that pandemic influenza had several differential features compared to historical seasons in the region. It arrived earlier, caused significantly higher number of outpatient consultations in children in most countries and followed west to east spread that was previously observed during some influenza seasons with dominant A (H3N2) ifluenza viruses. The results of this study help to understand the epidemiology of 2009 influenza pandemic and can be used for pandemic preparedness planning.

## Background

The world has recently experienced the first influenza pandemic of the 21st century that officially lasted 14 months from June 2009 to August 2010 [1]. While pandemic A(H1N1) 2009 influenza virus infections caused a wide spectrum of illness, it did have a significant effect on particular subpopulations such as pregnant women, persons with underlying conditions, and young adults who were at higher risk of developing severe disease [2,3]. Where outpatient illness is concerned, there is lack of data comparing the pandemic with previous seasonal influenza outbreaks. This paper aims to compare the timing, geographical spread and the community effect of 2009 influenza pandemic as compared to previous seasonal influenza outbreaks in WHO European Region.

The WHO European Region includes 53 Member States (MS) and covers the area from Western Europe to the Pacific coast of the Russian Federation, with a total population of over 800 million people. Influenza surveillance in the countries of the region is coordinated by the WHO European Influenza Network, EuroFlu [4] administered by the WHO Regional office for Europe.

Following its identification and emergence in North America in March and April 2009 [5], pandemic A(H1N1) 2009 influenza was reported to have spread to an additional five countries by 28 April including two countries in WHO European Region: The United Kingdom and Spain [6]. After that date, the number of reported cases in the Region rose rapidly, and 19 countries in the Region reported confirmed cases of pandemic influenza by the end of May 2009. A few countries in the region (Israel, Norway, United Kingdom, Sweden, and Malta) experienced peaks of influenza activity before usual influenza season, i.e. the week 40 of 2009.

Early reviews of pandemic data have suggested the highest attack rates in children, followed by young adults, with lower rates in persons over age 60 [7,8]. Studies comparing the rates of hospitalizations and deaths from pH1N1 to the average rates from seasonal influenza revealed a high burden of the disease in children and working population during the 2009-2010 influenza pandemic in different parts of the world [2,9]. However, to assess the magnitude of the 2009 influenza pandemic, it is also important to assess the burden of the disease associated with the outpatient illness. Larger numbers of milder cases of influenza impose higher demands on health care systems and contribute to economic costs related to work absenteeism [10,11]. However, there are scarce data on the effect of the 2009 influenza pandemic on outpatient illness with some studies focused exclusively on case-based surveillance [12] or presented clinical consultation rates of outpatient visits during the 2009-2010 pandemic influenza season and prior seasons without quantifying observed differences [13]. Since the general population had limited or no immunity against the novel A(H1N1) virus, we hypothesized that there would be increased outpatient consultation rates associated with influenza like illness (ILI) or acute respiratory infections (ARI) relative to previous seasons.

In order to better understand the epidemiology of pandemic influenza in WHO European Region, we also analyzed the timing and geographic spread of A(H1N1) virus in the Region in relation to those observed during previous influenza epidemics. In particular, it was expected that a novel virus would spread rapidly in a susceptible population resulting in an earlier influenza activity peak in the Region [14]. Next, the geographical spread of novel virus might not have followed west to east or south to north direction that is commonly observed in the Region [15].

We used existing influenza surveillance data in countries of WHO European region to answer the following research questions:

1. What was the timing of 2009-2010 pandemic influenza season compared to previous influenza seasons?

2. What was the direction of geographic spread of influenza virus during the pandemic season?

3. Were the total and age-specific outpatient consultation rates for ILI or ARI observed during the 2009 pandemic different from those observed during the seasonal influenza outbreaks?

## Methods

### Data collection

Data analyzed in this paper were abstracted from the WHO European Region database for influenza surveillance (Euroflu) [4] that collects data from 46 Member States of the WHO European Region. EuroFlu presents epidemiological and virological data that are collected by clinician networks and laboratory networks on a weekly basis. The clinician networks are represented by a stable group of general practitioners that cover a representative sample of the general population [16]. The primary care physicians report the weekly number of clinical cases of influenza-like illness (ILI) and/or acute respiratory infection (ARI) to a central registry and take respiratory specimens that are sent to a national reference laboratory for testing. This ensures that the clinical data reported by the sentinel physicians are validated by virological data on influenza. Some countries located in the central and eastern part of the Region have a universal surveillance system, meaning that all cases of ILI/ARI in countries with such surveillance systems are reported.

#### ILI and ARI case definitions

The case definitions of ILI and ARI used in different countries of the Region vary but share common criteria [17]. The general criteria for ILI are: sudden onset of fever > 38°C, with respiratory and systemic symptoms; the criteria for ARI are: sudden onset of respiratory symptoms, accompanied by fever and headache in the absence of other diagnosis. For countries reporting both ILI and ARI rates to EuroFlu, we selected the case definition that is being used for taking specimens for virological testing in a particular country. In addition, in some countries reporting both rates, ILI surveillance has been introduced relatively recently and no historical data for ILI are available for comparisons. For such countries, we selected ARI rates to enable comparison with historical data.

#### Selection of countries

For this paper we selected all countries in the WHO European region that have reported their weekly ILI or ARI rates to EuroFlu for the 2009-2010 pandemic influenza season and at least three past seasons. In addition, countries should have had clear influenza activity, *i.e*. in relation to historical data and with a defined peak of influenza activity, for at least 4 winter seasons including the pandemic season, to be included in the analysis.

#### Identification of peaks of influenza activity

The analyses presented in this paper are based on identification of influenza activity peak-weeks observed during the winter period, *i.e*. from week 40 to week 20. The peak weeks of influenza activity were selected by plotting the clinical and virological data available for each country. Normally, a week with the highest consultation rate was selected, and if two similar peaks were observed, a peak with a higher number of influenza virus detections was selected. If the clinical and virological activity was very low during a season, or if it was difficult to identify the peak week, no peak was selected, and the season was omitted from the analyses. The identification of peak weeks was undertaken independently by two researchers using the aforementioned rules, and any inconsistencies were resolved through discussion. For the Russian Federation, peaks were identified for its seven federal districts separately. For countries that observed summer and winter waves of pandemic influenza in the season 2009-2010, the data from winter wave were analyzed.

### Assessment of timing, duration, and geographic spread of influenza virus

To assess the timing of influenza activity in the Region, we calculated the median peak week of influenza activity in the Region for each of the seasons based on peak weeks of influenza activity in each of the included countries. Next, we plotted the median peak week of influenza activity in the Region, its interquartile range, and lower and higher confidence limits for the median for each season. The duration of influenza seasons in the Region was calculated by subtracting the earliest and the latest week of peak clinical activity observed in any country. As influenza activity starts several weeks before observing the first peak and lasts for some weeks after the last peak, we added four weeks to the length of the epidemic in the Region: 2 weeks before the earliest peak and 2 weeks after the latest peak [15].

The geographic spread of influenza activity was assessed by plotting the peak week of influenza activity in the countries against the longitude and latitude of the central point in each country. A squared correlation coefficient was computed to assess the relationship between longitude/latitude and the sequence of influenza activity peak weeks in different countries. We assessed the geographic spread of influenza virus during the winter period, *i.e*. from week 40/2009 to week 20/2010, as there were only 5 countries that experienced peaks of influenza activity before that period, making it impossible to analyze the course of peak activity in a small set of countries before week 40.

For the purpose of finding the appropriate geographic centre of a country, rounded longitude and latitude data were used, based on the Gazetteer of Conventional Names [18]. For the Russian Federation, the data were analyzed on a federal district level, i.e. the longitude, latitudes (of the federal capital city) and week of influenza peak activity in each of the seven districts were included in the analysis to ensure a detailed analysis of the geographic spread in this large country.

We assessed the geographic spread of influenza activity for the whole Region and in two subsets of the countries in the Region. For the subset analysis, we assessed the geographic spread in countries that are located to the west from the longitude line of 60 degrees east and in countries located to the west from the longitude line of 30 degrees east. The longitude of 60 degrees East was chosen as it marks the Ural Mountains holding the major part of the traditional physiographic boundary between Europe and Asia [19,20]. The longitude of 30 degrees East was chosen to investigate the geographical spread of influenza in the western part of the WHO European region [20], Majority of countries located in this part of the region are Member States of the European Union and/or are signatory States to the Schengen agreement [21]; meaning that persons in these countries can move freely from country to country, contributing to more intense interaction between populations of the countries located in this part of the Region.

### Assessing the effect of the pandemic on outpatient clinical consultation rates

To assess the effect of the pandemic on ILI/ARI clinical consultation rates, the weekly total and age-specific (where available) ILI or ARI rates per 100,000 population corresponding to the observed epidemic peak week as well as the four weeks preceding and four weeks following the peak weeks were abstracted for each country and season. We used simple linear regression models (with a single predictor variable being type of influenza season, i.e. seasonal versus pandemic) to compare clinical consultation rates for the 2009-2010 winter influenza season to the rates from seasonal influenza outbreaks in each of the included countries. The simple linear regression equation for a specific country for the total ILI or ARI rates was following: y = a + b*x, where "*y" *was the total ILI or ARI rate for a specific country; "*a" *was the average ILI or ARI rate observed in that country during the whole study period; "*b" *indicated how much the ILI or ARI rate observed during the pandemic season deviated from an average ILI or ARI rate in the study period; and "x" was a binomial variable indicating if a season was pandemic or not (0 = pandemic, 1 = seasonal outbreak). Therefore, if *b *was a positive number, it indicated that the ILI or ARI rates observed during the pandemic were higher in relation to previous seasons. Consequently, the negative value for *b *indicated lower ILI or ARI rates observed during the pandemic in a particular country in relation to historical seasons included in the analyses. Separate regression models were constructed for total and age-specific ILI/ARI rates in each country. Age-specific analyses were conducted for countries that provided age-specific data. Age groups used were 0-4, 5-14, 15-64 and over 65. We conducted all statistical analyses using SPSS version 17.0. (SPSS Statistics 17.0: Chicago)

## Results

**Figure 1:**
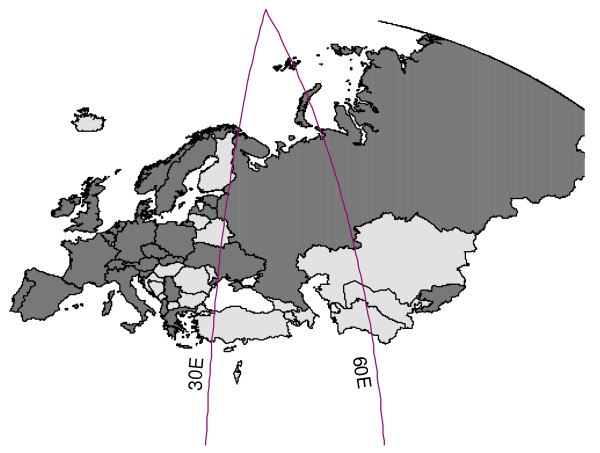
**Countries of the WHO European Region included in the analysis**. Dark grey color indicates the countries of the Region that are included in the analysis. Longitudes of 30 and 60 degrees east indicate cut off values for conducting the analysis of geographic spread in different subsets of countries.

**Figure 2:**
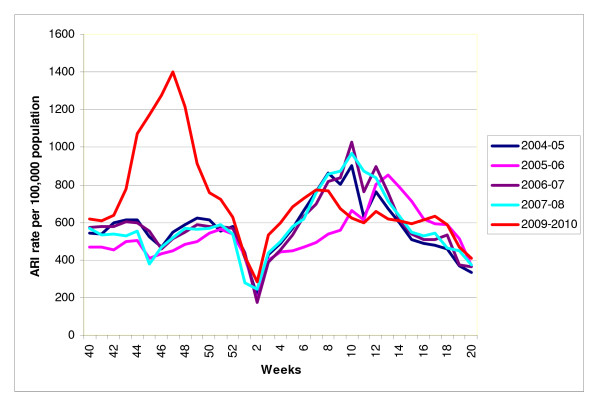
**Incidence of outpatient consultation rates for ARI in the Russian Federation in winter periods during the 2004-05 to 2009-2010 influenza seasons**.

Twenty eight countries in the WHO European Region provided their influenza surveillance for the last four or more years and were eligible for inclusion in the study (Figure 1). Twenty-four of these countries provided information on ILI rates, and 15 countries reported ARI rates as an epidemiological indicator of influenza activity in their countries (of these, 11 reported both ILI and ARI rates). We used ILI rate for the analyses in 20 countries and the ARI rate for the remaining eight countries (Table 1). Age-specific outpatient clinical consultation rates were provided by 20 out of 28 countries. The Figure 2 provides an example of ARI rates observed in the Russian Federation during six influenza seasons including the 2009-2010 pandemic season.

**Table 1:** Countries included in the analyses and description of data used

Countries	Data reported to EuroFlu	Data used in the analyses	Age specific data	N of seasons available*	N of seasons included **
Albania	ARI	ARI	no	7	7

Austria	ARI & ILI	ILI	yes	6	5

Belgium	ARI & ILI	ILI	yes	7	7

Czech Republic	ARI & ILI	ARI	yes	7	6

Denmark	ILI	ILI	yes	7	7

England	ARI & ILI	ILI	incomplete	7	6

Estonia	ARI &ILI	ILI	yes	5	4

France	ARI	ARI	yes	7	7

Germany	ARI	ARI	yes	7	6

Greece	ILI	ILI	no	5	5

Hungary	ILI	ILI	yes	5	5

Ireland	ILI	ILI	yes	7	7

Italy	ILI	ILI	incomplete	7	7

Kyrgyzstan	ARI	ARI	no	5	4

Latvia	ARI & ILI	ARI	yes	7	7

Netherlands	ILI	ILI	yes	7	7

Northern Ireland	ARI & ILI	ILI	yes	7	6

Norway	ILI	ILI	yes	5	5

Poland	ILI	ILI	yes	7	7

Portugal	ILI	ILI	yes	7	6

Russian Federation	ARI & ILI	ARI	yes	6	6

Serbia	ILI	ILI	yes	4	4

Slovakia	ARI & ILI	ILI	yes	7	7

Slovenia	ARI ILI	ILI	yes	7	7

Spain	ILI	ILI	yes	7	7

Sweden	ILI	ILI	no	5	5

Switzerland	ILI	ILI	no	7	7

Ukraine	ARI & ILI	ARI	no	7	7

ILI: Influenza like illness; ARI: Acute respiratory infection

* Availability of epidemiological data in EuroFlu from season 2003-04 onwards

** depends on reporting of data by countries to EuroFlu and the presence of an outbreak with a clearly defined peak in a given season

### Timing and duration of pandemic in the WHO European region

**Figure 3:**
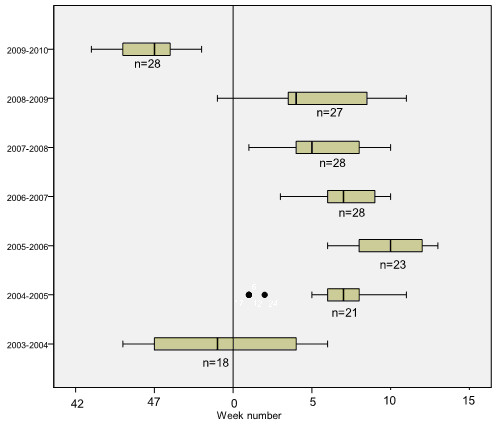
**Boxplots of influenza activity timing in the WHO European Region for the recent 7 influenza seasons**. The box plots present medians, interquartile range, and the lower and upper confidence of influenza activity peak week observed in the countries of the WHO European Region in 7 recent influenza seasons.

During the 2009-2010 pandemic influenza season, the median influenza activity peak in the Region was observed during the week 47. (Figure 3) There was a perfect synchronization of influenza activity peak across age groups during the pandemic 2009-2010 influenza season. In most countries the age-specific peak weeks coincided. In few countries, the peaks of influenza activity in children were observed one or two weeks earlier than in adults. The mean difference between the median week of influenza activity peak observed in pandemic versus the median of historical seasons was 10.5 weeks (95% CI 6.4-14.2). The overlapping lower and upper confidence limits for the pandemic 2009-2010 and 2003-04 seasons indicate that the timing of influenza peaks during these two seasons was not significantly different.

The duration of influenza activity in the Region during recent influenza seasons varied from 14 to 19 weeks. The duration of influenza activity during the pandemic season was 14 weeks, which is similar to the duration observed during the shortest historical seasons, *i.e*. 2005-06 and 2006-07. The 2003-04 and 2008-09 seasons were the longest seasons in the Region, both lasting 19 weeks.

### Geographic spread of pandemic A (H1N1) virus in the Region

During the winter wave of 2009-2010 influenza pandemic, the ILI and ARI consultation rates in the WHO European Region reached their peak between week 43/2010 England and Northern Ireland in the United Kingdom and week 50/2010 in Hungary. There was a moderate correlation between longitude of country midpoints and peak influenza activity in the western part of Europe (R2 = 0.282, *p *< 0.05), indicating a west-east spread of influenza (Table 2). However, when countries located in central and eastern parts of the WHO European region (Figure 1), *i.e*. east to longitude of 30 degrees east, were included in the analysis, the squared correlation coefficient indicated absence of the west to east spread pattern in these parts of the Region. (Table 2) The analysis of correlation between latitudes and influenza activity peaks did not show south-north spread in any part of the Region.

**Table 2:** Spread of pandemic (H1N1) virus in WHO European Region during 2008-09, 2009-2010, and 2010-2011 influenza seasons

Season	W-E R^2^	S-N R^2^	N of countries †
2010-2011			

Western part of the WHO European region §	0.244*	0.040	36

Western and central parts of WHO European region ‡	0.080	0.030	45

WHO European region	0.002	0.000	51

2009-2010			

Western part of the WHO European region §	0.282*	0.037	26

Western and central parts of WHO European region ‡	0.088	0.043	30

WHO European region	0.001	0.051	35

2008-2009			

Western part of the WHO European region §	0.731*	0.000	25

Western and central parts of WHO European region ‡	0.852*	0.020	34

WHO European region	0.330*	0.002	39

† Federal districts in the United Kingdom and the Russian Federation referred as separate countries in this analysis

*significant at 0.05 level

W-E west to east spread; S-N south to north spread; R^2^: squared correlation coefficient

§ countries located before longitude line of 30 degrees east

‡ countries located before longitude line of 60 degrees east

It is important to mention that the geographic spread of influenza viruses in the Region followed the same pattern in the first post pandemic, 2010-2011 influenza season, with significant west to east spread observed only in the western part of the region. This is in contrast to 2008-2009 season, when the west to east spread was observed in all parts of the region. (Table 2) During the 2009-2010 and 2010-2011 influenza season, the A(H1N1) pdm09 influenza virus was the (co) dominant virus, and in 2008-2009 influenza season, influenza A(H3N2) was the dominant virus in the Region.

### Effect of the pandemic on outpatient clinical consultation rates

In nine (32%) out of 28 countries, the total ILI or ARI consultation rates observed during the 2009-2010 pandemic season were significantly higher (*p *< 0.05) compared to the rates observed during previous influenza seasons (Table 3). The other countries experienced similar total rates of outpatient consultations during the pandemic compared to historical data.

**Table 3:** Comparison of total and age-specific ILI or ARI consultation rates observed during the pandemic in relation to previous influenza seasons

**Country **Ŧ	**Age specific and total regression coefficients **‡				
	
	0-4	5-14	15-64	Over 65	Total
**All ages affected**					

Norway	0.666*	0.667*	0.555*	0.325*	0.606*

**Children and working population affected**					

Ireland	0.815*	0.816*	0.641*	0.063	0.746*

Northern Ireland	0.638*	0.748*	0.465*	-0341*	0.588*

**Children affected**					

Russian Federation	0.540*	0.680*	0.436	-0.436	0.606*

Estonia	0.538*	0.515*	0.223	-0.072	0.407*

Netherlands	0.400*	0.414*	0.022	-0,286*	0.107

Spain	0.358*	0.488*	0.056	-0.147	0.234

Portugal	0.375*	0.520*	-0.179	-0.272*	-0.60

Denmark	0.271*	0.328*	0.115	-0.204	0.157

Latvia	0.296*	0.110	-0.203	-0.379*	-0.102

Austria	-0.052	0.345*	-0.123	-0.424*	-0.026

Slovenia	-0.071	0.353*	0.038	-0.033	0.133

**No effect**					

Belgium	0.034	0.184	-0.026	-0.216	0.121

Poland	0.047	0.115	0.052	-0.151	0.048

Czech Republic	-0.172	0.024	-0.253	-0.394*	-0.216

France	0,076	-0.193	-0,117	0.011	-0.057

Germany	-0.267	0,126	0.006	-0.305*	-0.053

Poland	-0.013	0.068	0.003	-0.203	-0.003

Serbia	-0.157	0.121	0.127	-0.472*	0.033

Slovakia	-0.304*	-0.186	-0.314*	-0.399*	-0.297*

Switzerland	n.a.	n.a.	n.a.	n.a.	**0.207**

Ukraine	n.a.	n.a.	n.a.	n.a.	**0.048**

Albania	n.a.	n.a.	n.a.	n.a.	**-0.122**

Italy	n.a.	n.a.	n.a.	n.a.	-0.282

**Significant overall effect**					

Sweden	n.a.	n.a.	n.a.	n.a.	0.358*

Greece	n.a.	n.a.	n.a.	n.a.	0.303*

Kyrgyzstan	n.a.	n.a.	n.a.	n.a.	0.332*

England	n.a.	n.a.	n.a.	n.a.	0.295*

The regression coefficients compare the total and age-specific ILI (influenza like illness) or ARI (acute respiratory infection) rates observed during the peak week of influenza activity, four weeks preceding the peak and four weeks following the peak in each of the countries

Ŧ Ranking is based on the regression coefficient for 0-4 age group

‡ a positive regression coefficient means that the rate observed during the pandemic was higher compared to the historical seasons; a negative regression coefficient means that the rate observed during the pandemic was lower compared to the historical seasons

*significant at *p *< 0.05

The analysis of age specific consultation rates in 20 countries with available age-specific data revealed a significant increase in outpatient consultation rates for the children aged 0 to 4 years in 10 (50%) countries, and for children aged 5 to 14 years in 11 (55%) countries (Table 3) An increase in consultation rates in children was also observed in some countries, *i.e*. Denmark, Netherlands, Spain, and Slovenia, even though the total consultation rates observed during pandemic were not significantly higher relative to historical seasons (Table 3). Outpatient consultation rates in populations aged over 65 were significantly lower during the pandemic season compared to historical data in 10 (50%) countries providing age-specific data.

## Discussion

Our study demonstrated similarities and differences in the 2009-2010 pandemic influenza season when compared to previous influenza seasons in the Region. During the 2009-2010 influenza season, influenza activity peaked in the Region much earlier than in five previous seasons. During the 2009-2010 season there were also significantly higher clinical consultation rates of ILI/ARI observed in younger populations. Similar to some of the previous seasons, the influenza virus followed a west to east direction in the western part of the Region, and the duration of the winter wave of the pandemic influenza was short, but similar to some other prior seasons.

The activity of pandemic influenza peaked very early in the Region most probably due to the emergence of the novel virus. This is consistent with the notion that susceptibility of the population may play a more important role that the other factors, *i.e*. absolute humidity [22,23], variation in vitamin D levels related to the amount of sunlight [24], and patterns of social mixing [25], associated with the seasonality of influenza outbreaks in temperate climates [26]. A similarly early peak in influenza season in the WHO European Region was observed during the 2003-04 season, when the Fujian strain of A(H3N2) influenza virus was the predominant virus circulating in Europe [27]. During this season, the pre-existed immunity in the general population was also limited due to a reassortment event in the A(H3N2) influenza virus [28,29].

The duration of epidemic period in the Region during pandemic influenza season was not shorter that during some other recent influenza seasons. This could be partially explained by the large size of the Region and a minimum time period required for the virus to spread to all countries. In addition, the secondary attack rate of pandemic A(H1N1) 2009 virus has been shown to be generally similar to the one usually observed for seasonal influenza viruses [30-32], contributing to the similar duration of influenza seasons.

We observed a moderate west- east spread of the influenza virus in the Western part of the Region. In the last eight years this direction was previously observed during 5 historical seasons with (co) dominant and generally more virulent A (H3N2) influenza virus [33]. The fact that this direction was observed only in the western part of the Region during the pandemic influenza season, could be explained by more extensive air traffic in this part of the region and its links with USA and Mexico where the first cases of pandemic influenza occurred and spread first to the Spain and United Kingdom in the Region [34]. In addition, the spread of influenza virus in the central and especially western parts of the region might be influenced by neighboring countries in the Middle East and Central Asia. We did not observe south to north spread of the virus in any parts of the Region; this direction of spread was previously found during a few, A(H3N2) dominated influenza seasons in Europe [15]. Monitoring the direction of geographic spread of influenza viruses in the region provides useful information for planning the vaccination, allocation of health care resources and public campaigns.

Although in most of the countries the total consultation rates were not higher when compared to historical seasons, children in 0-4 age (in 10 countries) and in 5-14 age groups (in 11 countries) experienced significantly higher consultation rates compared to past data. Apparently, the younger population had more limited immunity for the pandemic A(H1N1) influenza virus compared to 65+ group in our analyses, who experienced significantly lower consultation rates compared to previous influenza seasons. This could be explained by the previous exposure of older population to the similar A (H1N1) viruses that circulated before the 1957 pandemic [35-39]. It is remarkable that during all three previous influenza pandemics of 20^th^-century, older populations appeared to be more protected compared to children, probably due to a similar mechanism of the presence of pre-pandemic antibodies [40].

We found that some countries in the WHO European region experienced higher total or age-specific rates while others did not. The health seeking behavior of the population in different countries might have affected the rates of outpatient consultations for ILI/ARI. However, the percentage of sentinel respiratory specimens tested positive for influenza virus during the pandemic season in individual countries is very similar to the positivity rate observed prior the 2009 influenza pandemic [4]. This suggests that the differences observed between different countries are observed due to the stochastic nature of influenza spread. Differences in impact in different countries are typical for influenza pandemics [40], and could be explained by the heterogeneity in the degree of immunity in local populations to the pandemic influenza virus, as well as by differences in transmission factors such as local geographic conditions and patterns of social mixing.

### Strengths and limitations of the study

To our knowledge, this is the first study comparing the timing, geographic spread and outpatient clinical consultation rates for ILI or ARI of the 2009-2010 pandemic influenza season against historical data for a large number of countries. We used routine sentinel surveillance data, and it has been shown that both ILI and ARI consultations rates provided by countries with sentinel surveillance systems are reliable estimates of influenza activity, *e.g*. peaks of influenza activity based on ILI or ARI rates are well supported by virological confirmations [15]. Another strong point of the analysis is inclusion of data for 2003-04 influenza season, during which the predominant A(H3N2) virus caused higher consultation rates in children of 0-4 age in several countries [41].

Sentinel ILI/ARI surveillance data that were used for the current analyses, are collected systematically from a standard number of sites, thereby enhancing the comparability of the 2009-2010 data to those from historical seasons. Although three countries (Kyrgyzstan, the Russian Federation, and Ukraine) in these analyses provided non sentinel epidemiological data, considering that these countries have universal ARI surveillance systems that have been operating for many years, *i.e*. all ARI cases are being routinely reported, we expect the data provided by these countries to reliably represent influenza activity in their countries.

Although the case definitions for ILI or ARI used by countries are differing, it is important to highlight that for this analysis no between-country comparisons have been made. Instead, data from each of the included countries were compared over the time. When comparing outpatient consultation ILI or ARI rates in each of the countries, we assumed that there were no changes in health care systems of the countries that might have affected the consultation rates over the time. However, the ILI or ARI rates in some of the countries might have been influenced by media bias, i.e. attention that had been paid to the influenza pandemic by mass media could have affected the health seeking behavior resulting in higher consultation rates. Since we have based our analysis on the winter wave of the pandemic and we have excluded the ILI or ARI rates observed during the summer wave, when the consultation rates were most likely to be affected by media bias or panic [42], we expect less influence on health seeking behavior during the winter wave of the pandemic. On the other hand, a variety of prevention measures had been undertaken by countries during the pandemic influenza season, including vaccination, social distancing, school closures, antiviral treatment and prophylaxis that might have reduced the attack rates [43-45], and subsequently the outpatient consultation rates due to ILI or ARI. If these measures had not been undertaken, we might have observed a greater difference between pandemic and seasonal ILI/ARI rates in the countries of the Region.

The availability of surveillance systems for outpatient consultations rates allowed monitoring of influenza activity during the pandemic in the region and enabled this analysis to improve our understanding of epidemiology of the 2009 pandemic. However, there was no systematically collected data regarding hospitalization across the Region during the previous seasons to enable comparative analysis of risk factors for severe acute respiratory infections. Establishment of such systems in the countries of the WHO European Region is ongoing [46] and will improve greatly surveillance of future influenza outbreaks.

## Conclusion

In conclusion, we found that the 2009-2010 influenza pandemic in WHO European region had an epidemiological signature that was both similar and different to seasonal influenza epidemics since 2003 in the European Region. The 2009-2010 pandemic was similar length to previous seasonal epidemics and followed a west-east progression in western part of the Region that has been previously observed, albeit in seasons with dominant for A (H3N2) influenza virus. However, the 2009-2010 season arrived earlier than any season observed since the emergence of the "Fujian" H3 variant during 2003-04 influenza season, and had a uniquely strong impact on ILI/ARI clinical consultation rates among children. These results add to the growing body of knowledge about pandemic A (H1N1) 2009 that may be used for future pandemic preparedness across the Region.

## Competing interests

The authors declare that they have no competing interests.

## Authors' contributions

LM initiated the idea of the study, designed the study, selected the peaks, abstracted and recoded the data, conducted the statistical analysis, and wrote the first draft of the paper; WJP participated in the data collection, initiation and design of the study, and drafted the manuscript; PJ selected the peaks of influenza activity as a second researcher and drafted the manuscript; CSB, TM, and DP worked on the collection of data used in the manuscript and drafted the manuscript; JAM participated in the data collection, study design and drafted the manuscript critically for important intellectual content. All authors read and approved the final manuscript.

## Disclaimer

Maps used in this paper do not imply any opinions whatsoever on the part of WHO or its partners about the legal status of the countries and territories shown or about their borders.

## Pre-publication history

The pre-publication history for this paper can be accessed here:

http://www.biomedcentral.com/1471-2334/12/36/prepub
